# The Madness of Microbiome: Attempting To Find Consensus “Best Practice” for 16S Microbiome Studies

**DOI:** 10.1128/AEM.02627-17

**Published:** 2018-03-19

**Authors:** Jolinda Pollock, Laura Glendinning, Trong Wisedchanwet, Mick Watson

**Affiliations:** a Animal and Veterinary Sciences, Scotland's Rural College (SRUC), Edinburgh, United Kingdom; b The Roslin Institute and Royal (Dick) School of Veterinary Studies, University of Edinburgh, Edinburgh, United Kingdom; Chinese Academy of Sciences

**Keywords:** 16S RNA, DNA sequencing, microbiome

## Abstract

The development and continuous improvement of high-throughput sequencing platforms have stimulated interest in the study of complex microbial communities. Currently, the most popular sequencing approach to study microbial community composition and dynamics is targeted 16S rRNA gene metabarcoding. To prepare samples for sequencing, there are a variety of processing steps, each with the potential to introduce bias at the data analysis stage. In this short review, key information from the literature pertaining to each processing step is described, and consequently, general recommendations for future 16S rRNA gene metabarcoding experiments are made.

## INTRODUCTION

In recent years, the emergence of high-throughput sequencing platforms has revolutionized the study of complex microbial communities. Most commonly, marker genes (e.g., 16S rRNA and 18S rRNA genes) are amplified and sequenced, providing both qualitative and quantitative (i.e., relative abundance) data. However, the variety of methodologies which can be used to carry out marker gene analysis can be overwhelming. Each methodological stage, from sampling to data analysis, can introduce biases; such biases can skew data sets by introducing changes in the relative abundances observed, and they can affect the perception of community diversity. This short review includes key information from current literature on sample collection, sample storage and processing, and sequencing and data analysis, specifically for the study of bacterial communities using 16S rRNA gene metabarcoding. By collating fundamental research from each of these areas, we aim to try to ensure that scientists entering this field are better informed to make decisions on experimental design for 16S rRNA gene sequencing studies.

## SAMPLE COLLECTION

A sampling method is obviously dependent on sample type, and as such, the factors which may introduce bias will also vary between different types of microbiome studies. Clearly, study-specific concerns cannot be entirely covered in this review. However, the overarching factors which should be taken into account will be briefly covered in this section.

First, it is important to consider the proposed sampling site. Bacterial community composition varies even within a specific environment, for example, at different sites within the gastrointestinal tract (1) and the respiratory tract (2) and at different soil depths (3, 4). Since the magnitude of interindividual variation is very much dependent on sampling site (5), this can have implications for experimental design, specifically with regard to the number of subjects and the number of samples to be taken.

Second, there are conflicting results in the literature with regard to the variation introduced by different sample collection methodologies. For example, there have been attempts to replace invasive sampling with less invasive methods; however, significant differences have been found in microbial populations in comparisons of swab and biopsy samples from human intestines (6), breath condensate and lung brushings (7), and rumen fluid samples obtained via oral stomach tubing and a fistula (8). However, other works contradict these findings, with two studies showing no statistically significant differences when studying the rumen microbiota in cattle using a variety of sampling methods (9, 10). Additionally, no significant differences were evident in microbial composition in comparisons of sinonasal swabs and biopsy samples (11) and rectal swabs and stool samples (12). This kind of conflict in the literature is not uncommon, which leads to a lack of consensus and standardization.

A final consideration is whether samples should be homogenized, which appears to be most critical in studies on gut contents (8, 13) and on soil (14), since various microbial compositions have been observed in different stool fractions and in soils with various particle sizes. Although the literature is generally conflicting with regard to sampling methodology, it is important to consider that comparisons of data obtained using different approaches should be avoided.

## SAMPLE STORAGE

There is conflicting evidence on whether different storage conditions alone can have an impact on microbial community studies (15–18). It is often not practical to extract DNA from fresh samples; therefore, samples are generally stored for various durations prior to DNA extraction. Conventionally, it is assumed that rapid freezing to −80°C is best practice (18, 19), but this is not feasible for all study designs, for example, at remote sites where low-temperature storage is unavailable (20). Several studies have been carried out to assess the effects of storage conditions on study findings, which will be summarized in this section.

## FRESH VERSUS FROZEN SAMPLES

A couple of studies showed that freezing samples appeared to cause an increase in the Firmicutes-to-Bacteroidetes ratio in comparison with fresh samples (15, 19). Conversely, in a study by Fouhy et al., the only bacterial groups differentially expressed between fresh and snap-frozen fecal samples were the Faecalibacterium and Leuconostoc genera, with no significant differences being evident at the phylum or family level (18). No significant effects on microbial composition or diversity were observed in fecal samples refrigerated for 24 h (21) or 72 h (20) prior to DNA extraction.

The impact of storage duration has also been explored in various studies. Lauber et al. stored soil, feces, and skin samples at various temperatures and found that storage duration had no significant impact on overall bacterial community structure or diversity (17). In samples which were stored at −80°C for 2 years, a small number of changes in the microbial communities were observed, with increased abundances of lactobacilli and bacilli and a reduction in the total number of operational taxonomic units (OTUs) (for a definition of OTUs, please see Operational Taxonomic Unit Picking Methods, below). Using the data presented in the literature, processing fresh samples is generally the best approach, but when this is not possible, samples should be frozen for unequal amounts of time and processed in one batch or frozen for an equal amount of time and processed in multiple batches. The decision on how to proceed will be dependent on the duration of the sample collection phase and on the study design, but regardless of processing method, the storage duration and DNA extraction batch should be recorded to enable this to be taken into account during analysis.

## USE OF CRYOPROTECTANT

McKain et al. explored the effects of using a cryoprotectant (i.e., glycerol/phosphate-buffered saline) to store ruminal digesta samples and found that freezing samples without cryoprotectant caused a significant loss in Bacteroidetes when measured by 16S rRNA gene copy number by quantitative PCR (15). The authors consequently suggested that simply storing samples without a cryoprotectant and carrying out DNA extraction at a later date would impact downstream results with regard to archaeal and bacterial community composition. Choo et al. explored the effects of using several common preservative buffers (i.e., RNA*later*, OMNIgene.GUT, and Tris-EDTA) relative to samples stored dry at −80°C on fecal microbiota composition (20). Samples stored in the OMNIgene.GUT buffer diverged the least from the samples stored dry at −80°C, and the results obtained from the samples stored in Tris-EDTA diverged the most, with associated changes in relative abundances of biologically important bacterial groups, such as Escherichia-Shigella, Citrobacter, and Enterobacter. Additionally, RNA*later* has previously been shown to be unsuitable for the storage of samples subject to microbial community analysis, with samples stored in RNA*later* being the least similar to fresh samples and samples immediately frozen at −80°C (22, 23). Consequently, when considering the use of a cryoprotectant for storage, it is important to ensure that all samples are stored in the same manner.

## DNA EXTRACTION

During DNA extraction, it is important to consider that some microbial cells may be more resistant to lysis, such as bacterial endospores (24) and Gram-positive bacteria, which will have an impact on DNA extraction efficiency. The presence of inhibitors has also been found to directly impact DNA extraction efficiency (e.g., debris in environmental samples and organic matter in soil and feces) and can affect the efficiency of PCR downstream (reviewed in detail by Schrader et al. [25]). Common inhibitors include inorganic material (e.g., calcium ions), with the majority of inhibitors being organic matter, such as humic acid, bile salts, and polysaccharides. These issues will vary according to sample type; therefore, matrix-specific DNA extraction protocols should be optimized as part of a 16S rRNA gene metabarcoding experiment.

Besides phenol-chloroform DNA extraction methods, there are many commercial extraction kits available which incorporate mechanical and/or chemical/enzymatic lysis steps. Numerous authors have demonstrated that the abundances of specific bacterial groups vary in comparisons of different DNA extraction methodologies (8, 26–31). Specifically, variations in DNA yield and quality are obtained which can lead to different results in downstream analyses (28).

One key DNA extraction step which can introduce bias is the presence or absence of a mechanical lysis step. The inclusion of a bead-beating step has been linked to a higher DNA yield (8, 29, 32), higher bacterial diversity (29, 32), and more efficient extraction of DNA from Gram-positive and spore-forming bacteria (29, 33, 34). Consequently, some authors suggest that samples subjected to different DNA extraction methods are not comparable (8, 28, 35). Ultimately, the best approach is to utilize a method which extracts the highest yield and quality of DNA as possible without biasing the method toward particular bacterial taxa. To achieve this, the inclusion of a bead-beating step and prior optimization of the DNA extraction method to ensure optimal DNA yield and quality is recommended prior to carrying out 16S rRNA gene sequencing.

## SEQUENCING STRATEGY

### Library preparation.

Since the entire 16S rRNA gene cannot be sequenced using short-read second-generation sequencing platforms, a short region of the gene must be selected for PCR amplification and sequencing. There is currently no consensus on the most appropriate hypervariable region(s), and several studies have been carried out to determine the advantages and disadvantages of each. Importantly, the choice of hypervariable region(s) and the design of “universal” PCR primers have an effect on phylogenetic resolution (36–40). Indeed, no primer set is truly universal, with some commonly used 16S rRNA gene primers proving ineffective at amplifying biologically relevant bacteria (34, 41). Fouhy et al. explored the effects of primer choice (as well as DNA extraction and sequencing platform) on microbial composition data using a mock bacterial community and three primer sets (42), with differences in relative abundances and richness being observed.

Further biases can be introduced during PCR amplification due to the presence of PCR inhibitors (described in DNA Extraction, above), with the number of PCR cycles and the use of a high-fidelity polymerase (43) also having an impact on results. The formation of chimeras occurs in later PCR cycles when the highest concentration of incompletely extended primers compete with the original primers. Consequently, the potential for chimera formation can be reduced by lowering the number of PCR cycles (44). Previous work found that bacterial richness increased as the PCR cycle number increased (45, 46), but that cycle number had no significant effect on community structure (46). A lower number of PCR artifacts was found when using a high-fidelity polymerase than with a standard polymerase (43). The use of different polymerases has also been found to significantly affect PCR efficiencies for particular bacterial groups and overall bacterial community structure (46). Finally, the quantity of input DNA into a PCR has also been found to have a significant effect on observed bacterial community structure (31). In summary, there is not a “gold standard” hypervariable region for 16S sequencing, but it is important to consider that PCR reagents and PCR conditions should be optimized and kept consistent across a study.

### Sequencing platforms.

D'Amore et al. have studied the choice of sequencing platform most recently (47), and we refer the reader to that paper for a more-in-depth analysis. Illumina technology (primarily the MiSeq system) has become the most common sequencing platform for 16S rRNA gene metabarcoding. This is because the MiSeq system, in general, produces the most accurate longest reads and has a much higher throughput than the other platforms, which enables more samples to be sequenced at higher depth or lower cost. Indeed, while D'Amore et al. caution that the choice of sequencer depends on the question being asked, they note that the MiSeq system is likely to be the platform of choice in most cases. The Roche 454 sequencer was, for a long time, the platform most used for 16S studies. The potential longer reads of this technology have some advantages; however, it is now no longer available, as Roche retired the product in 2013. The 454 sequencer unfortunately suffered from an elevated error rate due to miscalling of homopolymers. The Ion Torrent and Ion Proton platforms are often available at low capital cost and produce data more quickly than the MiSeq system. However, the lower throughput and higher error rates mean that many researchers prefer to select the MiSeq system. While Illumina MiSeq offers the highest quality data, there are some reported problems with the platform. Illumina MiSeq error rates are often thought to be around 0.01%; however Kozich et al. showed that the actual error rates can be as high as 10% and recommend a complete overlap of 250-bp reads to correct for this (48). D'Amore et al. similarly showed library-dependent error rates in either read 1 or read 2 (but not the overlap) in MiSeq data, albeit at a lower rate (2 to 3%) (47). An improvement has been suggested to this, which involves a heterogeneity spacer that improves sequence diversity in the library (49).

PacBio and Oxford Nanopore technologies are able to sequence the full length of the 16S gene, which is of course very powerful. However, again error rates are an issue, in the range of 5 to 15% for both technologies, which can cause subsequent errors in downstream analysis. Despite the high error rate of long-read single-molecule sequencing systems (50–52), studies are beginning to appear to show their utility for 16S rRNA gene sequencing (53–56). For example, Schloss et al. were able to reduce the observed error rate for the V1 to V9 region from 0.69 to 0.027% for PacBio data, which is comparable to those for the Illumina, 454 and Ion Torrent systems (54). One of the drawbacks of the PacBio technology is its throughput, i.e., the number of samples that can be run on the platform simultaneously and at a reasonable cost is much lower than with the MiSeq system.

When planning a 16S sequencing study, three key considerations are the quality of sequence data, the cost of sequencing, and the length of generated reads, as detailed already in this section. A final factor is the number of samples which can be analyzed per sequencing run. When utilizing Illumina platforms, it is possible to use multiplexing strategies by implementation of unique single-indexed (57) or dual-indexed (48) (or barcoded) primers for library preparation. If the number of samples per run is increased, this is associated with a lower coverage (or number of sequences generated) per sample. If the coverage per sample is too low, the diversity of the microbial community being studied is likely to be underrepresented, as rarer members of the community are less likely to be detected. Therefore, guidance on the number of samples to be included per run should be obtained from small pilot studies (and observation of the resultant rarefaction curves) or published literature. In larger studies, more than one sequencing run may be required, and Caporaso et al. showed that the data were highly reproducible across sequencing lanes (57). The appropriate sequencing platform should be selected based upon the aims of the experiment and the error rates associated with the available platforms. Another key consideration is sequencing coverage and its relation to the number of samples to be run. When studying core members of a microbial community, lowering the amount of coverage by increasing the number of samples in a sequencing run may be an effective way to decrease costs. However, if rarer members of a community are of interest, lower sample numbers leading to increased coverage may be more appropriate.

## MOCK BACTERIAL COMMUNITIES

As part of 16S microbiome studies, it is useful to include a mock community control composed of predetermined ratios of DNA from a mixture of bacterial species. This not only allows the quantification of sequencing error (58) but also allows bias introduced during the sampling and library preparation processes to be identified (42, 47, 59, 60). For example, a mock community containing bacterial taxonomies which are of specific interest to the research group can be used to calculate whether these taxonomies are likely to be over- or underrepresented in samples. Similar to mock communities, spike-in standards can also be used to analyze bias and the reproducibility of methodologies (61). However, unlike mock communities, these standards are added directly to samples; therefore, quality control can be performed on a per-sample basis. However, there is a risk of crossover between the 16S rRNA gene sequences contained in the standards and those which may be found in samples. Consequently, care must be taken to select bacteria which are highly unlikely to occur in the samples of interest (62, 63) or which have been designed *in silico* and are dissimilar to sequences found in 16S databases (61).

There are a variety of sources which provide mock bacterial communities for use in research; however, some researchers choose to create their own mock communities in-house which more accurately reflect bacteria of interest and scientific importance. Preprepared bacterial communities are available in two different formats: DNA mock communities and whole-cell mock communities. The whole-cell mock communities are useful for establishing the efficiency of the DNA extraction step, whereas DNA mock communities will only assess the efficiency of PCR, clean-up, sequencing, and analysis steps. At the time of this writing, mock communities are available from the American Type Culture Collection (ATCC) and Zymo Research. When planning a 16S study, the inclusion of a mock community is strongly encouraged.

## ANALYSIS STRATEGY

### Comparing pipelines.

The analysis of large and complex 16S rRNA gene sequencing data sets requires the use of bioinformatic tools. There are many pipelines available to process and analyze 16S rRNA gene sequencing data, including the commonly used QIIME (64), MG-RAST (65), UPARSE (66) (https://www.drive5.com/usearch/manual/uparse_pipeline.html), and mothur (67). These packages contain sets of tools which facilitate the complete analysis of 16S rRNA gene data, from quality control to operational taxonomic unit (OTU) clustering. Where they differ is predominantly in their accessibility to those with limited computational knowledge and in the availability of documentation.

Nilakanta et al. compared seven different packages (mothur, QIIME, WATERS, RDPipeline, VAMPS, Genboree, and SnoWMan) and concluded that while all of these packages provide effective pipelines for 16S rRNA gene analysis, the extensive documentation which accompanies mothur and QIIME provides them with an advantage over the other packages (68). Plummer et al. analyzed a single data set using QIIME, mothur, and MG-RAST and found that there were few differences in the results with regard to taxonomic classification and diversity (69). However, there were differences in the ease of use of each of these packages and the time required for analysis, with QIIME being the quickest analysis package (approximately 1 h) and MG-RAST being the slowest (approximately 2 days, due to the need for manual quality control to remove multiple annotations of reads). The authors do state that although MG-RAST is the slowest analysis method, it is perhaps the most suitable package for users with no command line experience. Ultimately, the choice of analysis package will be made on the basis of the user's level of experience in bioinformatics and on the available resources at the user's host institution.

### Quality control, alignment, and taxonomic assignment.

It is essential to carry out quality filtering to remove DNA sequences which are of unexpected length, have long homopolymers, contain ambiguous bases, or do not align to the correct 16S rRNA gene region. Critically, sequences should then be screened for chimeras, as the presence of chimeric sequences can affect the interpretation of the final data set and could, for example, overinflate the perception of community diversity (70). A variety of tools have been developed to remove chimeric sequences, such as UCHIME (66) and Chimera Slayer (70). By including a mock bacterial community in a sequencing run, since the true sequences in these are known, the number of chimeric sequences can be calculated (58).

Sequences should then be aligned to a reference alignment or assigned to a suitable reference using a sequence classifier, such as the RDP Classifier, which uses a naive Bayesian approach based on 8-mers (71). Schloss showed that alignment quality can significantly impact diversity and can artificially inflate the number of bacterial OTUs, and advised against using alignments which do not take into account the secondary structure of the 16S gene (72). Of the three most commonly used alignments which are guided by secondary structure (i.e., Greengenes [73], RDP [74], and SILVA [75]), the Greengenes alignment was observed to be of poor quality, leading to significantly greater richness and diversity estimates.

Postalignment, sequences and OTUs are assigned taxonomies based upon their similarity to training sets, which are most commonly constructed from the Greengenes, RDP, and SILVA databases. Errors within these databases, caused by sequencing/PCR errors (76) or by the incorrect labeling of sequences (77), may lead to the misidentification of sequences. Another issue when relying on databases for taxonomic assignment is their bias toward bacteria which are clinically relevant in humans, meaning that researchers investigating nonhuman hosts or environmental samples may struggle to assign taxonomy to their sequences. For example, in a study of the honey bee gut microbiota, disagreement was found between the three databases listed above upon carrying out taxonomic assignments (78). At the genus level, the three databases concurred in their assignments for only 13% of sequences. The classification of sequences was improved by including bee-specific full-length 16S rRNA gene sequences in the training set, highlighting the need to include more representative sequences from a greater number of habitats.

This improvement in classification of sequences has been highlighted by Werner et al., who advised using the largest and most diverse database possible (79). This group also found that trimming the reference sequences to the primer region of interest improved classification depth. However, in a more extensively studied environment, such as the human intestine, Ritari et al. found that making a personalized reference database containing only bacterial species which were known to inhabit that niche led to an increase in lower-taxonomic-level assignments, probably due to less competition among sequences than with large databases (80).

## OPERATIONAL TAXONOMIC UNIT PICKING METHODS

Operational taxonomic units (OTUs) are the common currency of 16S or marker gene studies of microbiomes. The term was originally coined by Sokal and Sneath (81) and in its more general usage refers simply to groups of organisms that are closely related. There are two major methods for defining OTUs: reference-based and *de novo*. In reference-based clustering, sequences from a community are clustered against a known reference database, and in *de novo* clustering, the sequences are clustered according to pairwise distance measures. Reference-based OTUs are sometimes referred to as phylotypes (82). As with many areas of microbiome analysis, the evidence is mixed as to which of the two approaches is best. It has been found that *de novo* methods perform better in terms of the quality of OTU assignments (83), with another study showing that *de novo* OTUs were unstable (84). However, Westcott and Schloss (83) argued that OTUs can be stable yet still incorrect, and in particular, they showed that some reference-based techniques were sensitive to the order of sequences in the database. Sul et al. found that reference-based techniques produced results similar to those with *de novo* methods, with the added benefit of low computational overheads and the ability to compare data sets from different variable regions (85). Indeed, perhaps the major difference between reference- and *de novo*-based methods is that *de novo*-based methods have a significantly greater computational overhead, with the need to compare every sequence to every other sequence in its most naive form.

Even within clustering tools, the choice of parameters has been shown to have a critical impact on the results. While a threshold of 97% has become standard, Patin et al. have shown that 16S rRNA gene sequences as similar as 99% can represent functionally distinct microorganisms, which means that functionally diverse species would be clustered at the 97% threshold (86). However, that may rely on accurate sequences, and if those do not exist, the 97% threshold can help avoid an overestimation of biodiversity (87). Susceptibility to differing parameters may also be pipeline dependent (88). Given the controversy and potential biases of clustering sequences, some have suggested methods and models for using individual sequences to represent OTUs (i.e., remove the clustering step entirely) (89–92).

## CORRECTING FOR GENE COPY NUMBER

Different bacterial species also have various copy numbers of the 16S rRNA gene (93, 94), which can lead to misinterpretations in comparisons of the abundance of bacterial OTUs or attempts to construct a “true” description of the microbial community within a sample (95). It is unusual in 16S rRNA gene studies to have an accurate knowledge of the copy numbers for all identified OTUs. Therefore, tools have been developed which seek to correct for copy number variation using sequence databases and phylogenetic information to give a more accurate picture of the relative abundances of these OTUs. These include Copyrighter (96), rrNDB (93), functions in the picante R package and pplacer (97), and part of the PICRUSt package (98).

As these techniques are reliant on databases, the same problems are present as for taxonomic identification. Principally, lesser-studied bacterial taxonomies are less likely to be represented. It is also important to note that in comparisons of OTUs between samples rather than within a sample (e.g., in comparisons of treatment effects), the impact of copy number variation is reduced, as the under- or overrepresentation of OTUs would be consistent across samples as long as the same methodology had been used.

## CONTAMINATION ISSUES

Microbial DNA contamination arising from DNA extraction kits, PCR reagents, and the lab environment may have a particularly large effect when studying low-microbial-biomass samples. Salter et al. found that contamination in DNA extraction kits not only varied by manufacturer but by individual lot, and samples processed in separate laboratories contained different types of contaminating DNA (99). This lack of predictability led the authors to suggest that “negative” (or reagent-only) controls should be run alongside samples in all 16S rRNA gene metabarcoding studies. If reagent-only controls are not included, this can lead to a misinterpretation of results. When Salter et al. analyzed a data set comparing nasopharyngeal microbiota samples from children at two time points, they found that while the time points appeared to cluster separately, this effect was mainly due to bias caused by contamination from the extraction kits used. Randomization of samples prior to processing may help avoid the introduction of this type of bias. Contamination could also lead to the false identification of microbial communities where they do not in fact exist (100) and could affect our understanding of which bacteria are relevant in clinical samples (101).

The amplification of background contaminants from PCR reagents could perhaps be avoided via the use of primer-extension PCR (102), but this would have no effect on contamination originating from other sources. Several methods have been suggested to remove contaminating DNA from reagents and the lab environment, including UV and gamma radiation (103–107); DNA intercalation by 8-methoxypsoralen, ethidium monoazide, and propidium monoazide (104, 106–108); enzymatic treatments (105–107, 109–111); silica-based membrane filtration (112); CsCl_2_ density gradient centrifugation (111); and bleach/copper-bis-(phenanthroline)-sulfate/H_2_O_2_ (CoPA) solution treatment (105). These methods have shown varied effects on contamination levels and PCR sensitivity, and the inclusion of reagent-only controls alongside these decontamination measures is still recommended.

What should be done with sequencing data from reagent-only controls is still under debate. It is often not appropriate to simply remove all of the bacterial OTUs found in controls, as these may overlap OTUs which can genuinely be found in samples (108). Other methods have been suggested which take into account the abundance of OTUs to predict the likelihood of sequence reads having originated from contamination. These include adapting the neutral community model (12) and combining quantitative PCR data with OTU relative abundance data to compare the absolute abundances of contaminating OTUs in controls and samples (113). However, the field is rapidly reaching consensus that, due to contamination issues, not including reagent-only controls can negatively impact the quality control of sequence data. When planning a 16S study, the inclusion of reagent-only controls (i.e., DNA extraction kit and PCR controls) is advised.

## CONCLUSIONS

The study of complex microbial communities using high-throughput sequencing platforms has allowed a better understanding of a variety of biological systems and the impact of various conditions (e.g., disease states) on the host microbiome. Looking at the literature, it is clear that bias can be introduced into microbiota studies at all methodological stages from sampling to bioinformatic analysis. While the variety of different 16S rRNA gene metabarcoding methodologies might seem overwhelming, the main factor to keep in mind when designing a microbiota study is consistency. It is paramount to use consistent methodology throughout a study to minimize potential biases which could lead to spurious results.

The volume of studies attempting to define best practice for various stages of the microbiome experimental process is large, and we cover only some of the literature in this review. Unfortunately, as can be seen, there is little consensus, and further studies are unlikely to find any. The reality is that many of the biases described in this review are context and environment specific, and while individual studies may be true within their context, their conclusions may not be transferable to other studies. Clearly, with biases possible at every step, a good experimental design is essential. Recording and publication of all experimental metadata are essential for understanding microbiome studies, and unfortunately, many currently published studies lack these data.

Trying to find consensus in the literature is challenging, with many studies producing conflicting evidence about the effects of various steps in the experimental process. It is therefore essential that consistency is maintained within a study, and there must be an acceptance that comparisons between studies may not be possible.

In summary, we recommend extracting DNA from fresh samples if possible; if not, samples should be stored in a consistent manner (i.e., at the same temperature, for the same duration, and with or without cryoprotectant) with appropriate metadata being recorded. The use of a mechanical lysis step is recommended to minimize potential biases due to some microbial cells being more resistant to lysis. The selection of appropriate primers should be made after careful consideration of the literature, but it is important to note that even universal primers will not amplify all bacteria in a given sample. Sequencing both mock bacterial communities and “negative”/reagent-only controls is important for determining background contamination and sequencing error rate, and it should be included at least for each sequencing run and, even better, for every batch of commercial reagents/kits. To reduce the chance of OTU inflation caused by sequencing errors, consider complete overlap of MiSeq reads, which translates as targeting a single hypervariable region. Finally, and to reiterate, record every aspect of your experiment and report it in the methods section, and remember that the critical consideration is consistency in methodology at each stage.
